# Intraoperative Remifentanil Dosage in Surgery for Adolescent Idiopathic Scoliosis Does Not Increase Postoperative Opioid Consumption When Combined With Epidural Analgesia: A Retrospective Cohort Study

**DOI:** 10.7759/cureus.17361

**Published:** 2021-08-22

**Authors:** Yoshitaka Aoki, Hiroki Iwata, Chieko Akinaga, Yuki Shiko, Yohei Kawasaki, Kensuke Kobayashi, Hiroki Nozawa, Hiroyuki Kinoshita, Yoshiki Nakajima

**Affiliations:** 1 Department of Anesthesiology and Intensive Care Medicine, Hamamatsu University School of Medicine, Hamamatsu, JPN; 2 Clinical Research Center, Chiba University Hospital, Chiba, JPN; 3 Department of Anesthesiology, Seirei Mikatahara General Hospital, Hamamatsu, JPN

**Keywords:** remifentanil, hyperalgesia, opioid, scoliosis, epidural analgesia

## Abstract

Background

In adults, high-dose remifentanil during surgery has been reported to increase postoperative opioid consumption, but this has not been well documented in children. Multimodal analgesia is recommended in the perioperative period for adolescent idiopathic scoliosis (AIS), but no report has examined opioid consumption under epidural analgesia, which is one of the most common types of analgesia.

Aims

To investigate the association between intraoperative remifentanil dosage and postoperative opioid consumption in AIS in the setting of combined epidural analgesia for postoperative multimodal analgesia.

Methods

In this retrospective cohort study, patients aged 10-18 years who underwent surgery for scoliosis and epidural analgesia for postoperative pain between July 2012 and April 2019 were included. The primary endpoint was the association between intraoperative cumulative weight-adjusted remifentanil dosage and logarithmic transformation of cumulative weight-adjusted fentanyl consumption in the intensive care unit (ICU). Nonopioid analgesics were investigated as secondary endpoints. An epidural catheter was inserted by the surgeon intraoperatively, and a local anesthetic was administered at the end of the surgery. Multivariate linear regression analysis with adjustment for confounders was performed for all analyses.

Results

In total, 142 patients were included, and the median intraoperative remifentanil dosage for all patients was 0.27 (interquartile range, 0.24-0.34) µg/kg/min. No association was observed between cumulative weight-adjusted intraoperative dosage of remifentanil and fentanyl, even after adjusting for potential confounders (slope = −1.25; 95% confidence interval [CI], −4.35 to 1.85; P = 0.43). No association was observed between nonopioid analgesic use and intraoperative remifentanil dosage.

Conclusion

No association was noted between remifentanil dosage during surgery for AIS and postoperative opioid consumption with epidural analgesia. However, this study has limitations due to its retrospective design; thus, further prospective studies are warranted.

## Introduction

Surgery for adolescent idiopathic scoliosis (AIS) is challenging for anesthesiologists. Motor-evoked potentials (MEPs) must be monitored to avoid the risk of intraoperative motor dysfunction; only propofol and opioids are recommended for management, and no muscle relaxants can be administered [1,2]. Furthermore, because of the highly invasive nature of the surgery, intraoperative opioid dosages are inevitably high owing to concerns about acute opioid tolerance and hyperalgesia. A multimodal analgesic approach was reported to result in good outcomes [3] including epidural analgesia [4-6].

Remifentanil is an ultra-short-acting opioid that is the mainstay of analgesia for highly invasive surgeries because it induces a deep analgesic state without accumulating in tissues [7]. Remifentanil, which does not affect MEP [8], is the basis for analgesia during surgery for AIS. Intraoperative remifentanil dosages of >0.25 µg/kg/min are associated with increased postoperative opioid consumption, whereas dosages of >0.2 µg/kg/min indicate hyperalgesia characterized by low mechanical/pressure/cold/pain thresholds [9]. Previous studies have reported conflicting results on the association between remifentanil and hyperalgesia in surgery for AIS [10-12]. Calvin et al. reported no correlation between remifentanil dosage and postoperative opioid consumption during surgery for AIS [12]; however, this may not be a definitive conclusion owing to the small sample size and racial differences in their study [13]. Furthermore, they did not use epidural analgesia for postoperative multimodal analgesia.

We hypothesized that intraoperative remifentanil dosage is correlated with postoperative opioid consumption in AIS, even when postoperative epidural analgesia is used. This study was conducted to evaluate the association between intraoperative remifentanil dosage and postoperative opioid consumption and nonopioid analgesic consumption in the intensive care unit (ICU) in patients with AIS.

## Materials and methods

Study design and oversight

This retrospective cohort study was conducted at Hamamatsu University Hospital (Shizuoka, Japan) between July 2012 and April 2019. The study protocol was approved (19-194) by the Ethics Review Board of Hamamatsu University School of Medicine. Because of the study’s retrospective design and the absence of follow-up, the Ethics Review Board waived the requirement for written informed consent. This study was conducted according to the Strengthening the Reporting of Observational Studies in Epidemiology checklist [14] and complied with the 1964 Declaration of Helsinki and its later amendments.

Patients

We included patients who underwent surgery for scoliosis on the basis of the diagnosis of AIS and who were admitted to the ICU postoperatively. Following the definition of adolescence [15], the age of the patients was limited to 10-18 years. The exclusion criteria were as follows: (i) patients who used inhalational anesthetics, even for a short period, intraoperatively; (ii) patients who were not monitored for MEPs; (iii) patients who were not admitted to the ICU postoperatively; (iv) patients who had not received remifentanil intraoperatively; (v) patients who had a previous diagnosis of opioid dependence or alcohol abuse; (vi) patients who had received opioids for preoperative chronic pain; and (vii) patients who could not communicate because of psychiatric disorders or intubation upon ICU admission. In addition, patients who did not receive epidural analgesia were excluded from the analysis to ensure consistency in the analgesic background of the included patients.

Data collection

We selected patients on the basis of surgical and anesthesia data from the ERGA Anesthesia Systems (Suzuyo System Technology, Tokyo, Japan). The inclusion/exclusion criteria were manually checked by at least two independent authors.

Age, sex, body mass index (BMI), American Society of Anesthesiologists class, number of surgically fixed intervertebral vertebrae, operative time, anesthesia time, transfusion volume, urine volume, blood loss, intraoperative fentanyl use, intraoperative remifentanil use, intraoperative analgesia (acetaminophen and nonsteroidal anti-inflammatory drugs [NSAIDs]) use, and intraoperative epidural catheter insertion by surgeons were retrieved from the ERGA system. The intraoperative weight-adjusted remifentanil dosage (mg/kg) was calculated from the total remifentanil dosage divided by the patient’s weight immediately before the surgery. The data in the ICU, total opioid consumption, nonopioid analgesics (i.e., acetaminophen, NSAIDs, and dexmedetomidine), and length of ICU stay were collected from Prime GAIA (Nihon Kohden, Tokyo, Japan).

A multimodal approach to perioperative pain for AIS

In our institution, as a rule, intraoperative pain control consisted mainly of continuous remifentanil administration, with supplemental bolus fentanyl, acetaminophen, and NSAIDs administration. An epidural catheter is inserted by the surgeon during wound closure and a continuous epidural infusion is started. The drugs for epidural analgesia were preordered according to the operating surgeon’s preference, and the anesthesiologist or intensivist was not involved in the choice of drugs for epidural analgesia. The anesthesiologist initiates 15 µg/h of fentanyl continuous intravenous patient-controlled analgesia (IV-PCA) at the end of the surgery and then uses 15 µg of fentanyl PCA every 10 minutes of lock time if the patient feels pain in the ICU. The intensivist assesses the patient’s pain complaints and numerical rating scale (NRS) and adjusts the dosage of continuous fentanyl administered by a syringe pump. The intensivist may also administer a bolus dose of acetaminophen or NSAID as needed. Dexmedetomidine is also administered for sedation and analgesia and is increased or decreased as needed on the basis of the patient’s Richmond Agitation-Sedation Scale and NRS. If the patient has nausea and vomiting, fentanyl is reduced or discontinued, and other analgesics are used as the source of primary analgesia. In the present study, multimodal analgesia was continued until the patient was discharged from the ICU.

Outcomes and variables

The primary outcome was the association between intraoperative cumulative weight-adjusted remifentanil dosage and logarithmic transformation of cumulative weight-adjusted opioid consumption in the ICU, based on previous studies [12,16]. In this study, fentanyl was the only opioid consumed in the ICU. Therefore, the primary endpoint was total fentanyl consumption calculated by converting IV-PCA fentanyl concentration and continuous intravenous fentanyl concentration. Finally, multivariate linear regression analysis was performed to examine the factors associated with remifentanil dosage during surgery. In other analgesic adjuncts, doses of acetaminophen and NSAIDs and the total dose of dexmedetomidine administered in the ICU were calculated and established as secondary outcomes.

Covariates

The following covariates were used to adjust for confounders on the basis of previous studies and clinical significance [12]: age, sex, BMI, operative time, number of fixed vertebrae, intraoperative blood loss, intraoperative fentanyl dose, intraoperative acetaminophen dose, and intraoperative NSAID dose. Furthermore, the potency of different local anesthetics in epidural analgesia was converted to lidocaine and set as a confounding factor [17,18]. These covariates were forced in, and multivariate linear regression analyses were performed on all analyses of secondary outcomes in addition to those of the primary outcome.

Statistical analyses

Medians and interquartile ranges (IQRs) of continuous data and numbers and percentages (%) of categorical data were calculated. The association between the total dose of intraoperative weight-adjusted remifentanil and logarithmic transformation of postoperative weight-adjusted fentanyl consumption in the ICU was assessed using univariate and multivariate linear regression analyses. For secondary outcomes, multivariate linear regression analysis using the same confounder as the primary outcome was performed. P < 0.05 denoted statistical significance in all analyses. Statistical procedures were performed using SAS v9.4 (SAS Institute, Cary, NC, USA).

## Results

Selection and characteristics of patients

**Figure 1:**
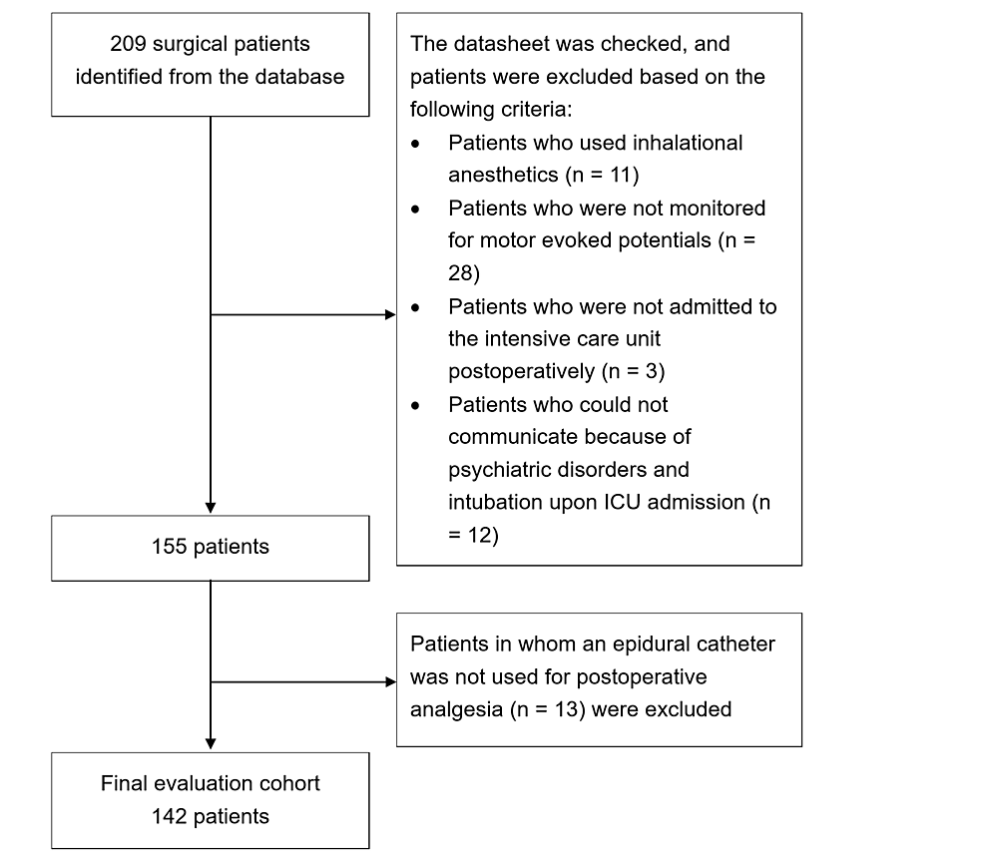
Study flowchart ICU: intensive care unit.

During the study period, 209 patients were admitted to the ICU at Hamamatsu University Hospital after surgery for AIS. After excluding patients as per the exclusion criteria, 142 individuals were included in the study (Figure 1). Table 1 presents the characteristics of the enrolled patients. The median weight-adjusted remifentanil dosage was 0.11 (IQR, 0.09-0.14) mg/kg, and the median intraoperative remifentanil dosage for all patients was 0.27 (IQR, 0.24-0.34) µg/kg/min. The only opioid administered in the ICU was fentanyl; thus, opioid consumption was expressed in fentanyl equivalents. Median weight-adjusted fentanyl consumption in the ICU was 21.7 (IQR, 13.9-32.4) µg/kg. There were no patients with motor dysfunction, but there were 14 patients with false-positive MEP.

**Table 1:** Baseline characteristics and outcomes of patients BMI: body mass index, ASA: American Society of Anesthesiologists, NSAIDs: nonsteroidal anti-inflammatory drugs, ICU: intensive care unit. Values given are numbers (column %) or median (interquartile range).

Clinical characteristic	
Age (years)	14 (13–16)
Female sex	123 (86.6%)
BMI (kg/m^2^)	18.8 (17.8–20.6)
ASA class	
Ⅰ	85 (59.9%)
Ⅱ	56 (39.6%)
Ⅲ	1 (0.7%)
Number of surgically fixed intervertebral vertebrae	10 (9–11)
Surgery time (hours)	4.5 (3.8–5.0)
Anesthesia time (hours)	6.5 (5.8–7.2)
Total dose of remifentanil (mg/kg)	0.11 (0.09–0.14)
Dose of remifentanil (μg/kg/min)	0.27 (0.24–0.34)
Total dose of propofol (mg/kg)	31.9 (28.3–36.3)
Fentanyl	142 (100%)
Dose of fentanyl (µg/kg)	10.5 (7.6–13.9)
Acetaminophen	84 (59.2%)
Dose of acetaminophen (mg/kg)	14.6 (0–19.6)
NSAIDs	40 (28.2%)
Dose of NSAIDs (mg/kg)	0 (0–0.9)
Infusion volume (mL)	2550 (2063–3038)
Urine volume (mL)	730 (446–998)
Blood loss (mL)	439 (252–685)
Transfusion volume (mL)	
Autologous blood collected preoperatively	400 (27.5–400)
Autologous blood recovered intraoperatively	107 (0–158)
Length of ICU stay (hours)	19.5 (18–21)
Postoperative epidural analgesia	
Bupivacaine	89 (62.7%)
Dose of bupivacaine (mg)	118 (74.5–149)
Levobupivacaine	27 (19%)
Dose of levobupivacaine (mg)	91.6 (75.3–170)
Mepivacaine	22 (15.5%)
Dose of mepivacaine (mg)	285 (260–309)
Ropivacaine	4 (2.8%)
Dose of ropivacaine (mg)	155 (102–217)
Outcomes	
Fentanyl consumption (µg/kg)	21.7 (13.9–32.4)
Acetaminophen dose (mg/kg)	0 (0–15.6)
NSAIDs dose (mg/kg)	0 (0–0)
Dexmedetomidine dose (µg/kg)	5.0 (3.7–7.4)

Primary outcome

**Figure 2:**
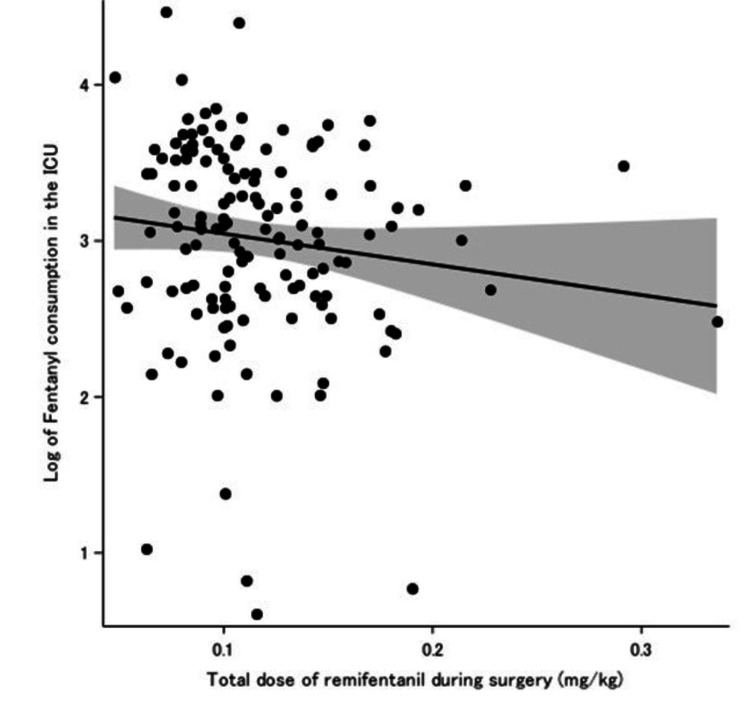
Univariate linear regression analysis comparing total dosage of intraoperative weight-adjusted remifentanil and logarithmic transformation of the postoperative weight-adjusted fentanyl consumption in the ICU The gray zone indicates the 95% confidence band. No correlation was found between total intraoperative remifentanil dosage and fentanyl consumption in the ICU (slope = −1.97; 95% confidence interval, −4.49 to 0.55; P = 0.13). ICU: intensive care unit.

Figure 2 presents the univariate linear regression analysis comparing the total dose of intraoperative weight-adjusted remifentanil and the logarithmic transformation of postoperative weight-adjusted fentanyl consumption in the ICU. After adjusting for potential confounders, the multivariate linear regression analysis result was consistent with that of the univariate linear regression analysis (slope = −1.25; 95% CI: −4.35 to 1.85; P = 0.43). Multivariate linear regression analysis revealed that female sex and total propofol dosage might correlate with the logarithmic transformation of fentanyl consumption in the ICU (Table 2). As supplementary material, Table 3 showed a list of potencies of various local anesthetics administered for epidural analgesia, converted to lidocaine.

**Table 2:** Multivariate linear regression analysis of the logarithmic transformation of fentanyl consumption in the intensive care unit BMI: body mass index, NSAIDs: nonsteroidal anti-inflammatory drugs, ASA: American Society of Anesthesiologists.

Variables	Regression coefficient	Standard error	95% confidence interval		P-value
Total dose of remifentanil (mg/kg)	−1.98	1.60	−5.15	1.19	0.22
Age (years)	0.007	0.04	−0.07	0.08	0.85
Female sex (ref. male)	0.45	0.19	0.07	0.82	0.019
BMI (kg/m^2^)	−0.024	0.023	−0.07	0.022	0.30
Lidocaine equivalent epidural analgesia (mg/h)	-0.076	0.056	-0.19	0.035	0.18
Acetaminophen (mg/kg)	0.009	0.006	−0.004	0.021	0.17
NSAIDs (mg/kg)	0.10	0.11	−0.12	0.33	0.37
Surgery time (hours)	-0.051	0.24	−0.52	0.42	0.83
Intraoperative blood loss (mL)	−0.000009	0.00018	−0.00037	0.00035	0.96
Fentanyl (µg/kg)	−0.016	0.015	−0.045	0.013	0.27
Number of surgically fixed intervertebral vertebrae	0.037	0.036	−0.034	0.11	0.31
Anesthesia time (hours)	−0.078	0.22	−0.51	0.36	0.72
Total dose of propofol (mg/kg)	0.025	0.012	0.0003	0.05	0.047
ASA	0.044	0.11	-0.18	0.27	0.70

**Table 3:** Conversion formula for local anesthetics

Local anesthetics	Potency
Lidocaine	2
Mepivacaine	2
Bupivacaine	8
Levobupivacaine	8
Ropivacaine	6

Secondary outcomes

Multivariate linear regression analysis using the same confounders as the primary endpoint revealed no correlation of intraoperative remifentanil dosage with total acetaminophen dosage (slope = −17.1; 95% CI, −64.0 to 29.8; P = 0.47), total NSAID dosage (slope = −1.01; 95% CI, −2.61 to 0.60; P = 0.22), and total dexmedetomidine dosage (slope = 6.18; 95% CI, −8.24 to 20.6; P = 0.40).

## Discussion

This study was conducted to determine whether intraoperative remifentanil dosage is correlated with postoperative opioid consumption in patients undergoing surgery for AIS and treated with postoperative epidural analgesia. In the 142 patients monitored for MEP during total intravenous anesthesia, remifentanil was administered at a median dosage of 0.27 µg/kg/min during surgery, which was not associated with postoperative opioid consumption in the ICU. Additionally, the dosages of acetaminophen, NSAIDs, and dexmedetomidine for postoperative multimodal analgesia did not increase. On basis of the results, it is likely that remifentanil dosage during surgery for AIS is not correlated with postoperative opioid consumption.

We emphasize that the novelty of this study lies in the use of postoperative epidural analgesia as a multimodal analgesia for AIS. Although epidural analgesia is effective in reducing opioid consumption after spine surgery [4], it is unclear whether it reduces acute tolerance and hyperalgesia in the ICU. Despite the limitations of different drug contents of continuous epidural analgesia, the effect of epidural analgesia was modulated as a confounding factor that was determined before ICU admission. This study is the first to investigate acute tolerance and hyperalgesia of opioids in patients who received epidural analgesia.

Our results showed that intraoperative remifentanil dosage was not associated with postoperative opioid consumption in adolescents. The results of this study conform to those of a recent study by Calvin et al. [12], which reported no association between total intraoperative remifentanil dosage and cumulative opioid consumption up to 72 hours postoperatively. The difference between our study and theirs is that they investigated opioid consumption up to 72 hours postoperatively, whereas we focused on a limited timeframe within the ICU. However, considering that hyperalgesia peaked at four hours after ceasing remifentanil infusion in a study on human volunteers [19], we set the duration of ICU stay to reliably monitor the patients. The strengths of our study are as follows: the sample size of this study (n = 142) was approximately twice that of their study (n = 78) [12], we used postoperative epidural analgesia as a part of multimodal analgesia, and we focused on postoperative nonopioid analgesic consumption. The concordance of these results is noteworthy because racial differences in opioid sensitivity [13] and postoperative orthopedic pain vary from country to country. The agreement between the results of their study and ours suggests that the finding of no correlation between remifentanil dosage during surgery for AIS and postoperative opioid consumption is valid.

This study’s multivariate linear regression analysis revealed that female sex and propofol dose were associated with postoperative opioid consumption. In previous studies, women have been reported to experience more postoperative pain [20]. Propofol has analgesic effects owing to N-methyl-D-aspartate (NMDA) receptor antagonism [21]. This result of female sex and propofol dose being associated with postoperative opioid consumption was only a secondary endpoint and should be investigated further.

This study revealed no association between intraoperative remifentanil dosage and postoperative acetaminophen, NSAID, and dexmedetomidine dosages in the ICU. The mechanism of acute opioid tolerance is not precise, but it has been suggested that the expression, transport, and function of NMDA and non-NMDA glutamate receptors in the dorsal horn play a central role in it [22]. Ketamine, an NMDA antagonist, has been associated with decreased acute opioid tolerance [23]. However, ketamine is not commonly used in Japan because it has many side effects (e.g., increased blood pressure and heart rate, hallucinations, and delirium) [24]. Acetaminophen, NSAIDs, and dexmedetomidine, selected in this study, are commonly selected as postoperative multimodal analgesics for AIS and to decrease opioid consumption [4-6]. We assumed that in adolescents with postoperative nausea and vomiting, which are side effects of opioids [25], nonopioid analgesic dosages would be increased rather than increasing opioid dosage. However, the results of this study showed that nonopioid analgesics were not correlated with intraoperative remifentanil dosage in AIS.

This cohort study had several limitations. First, it was a retrospective study conducted at a single institution. No strict protocol exists for increasing or decreasing intraoperative remifentanil and administering opioids and other analgesics in the ICU; this is at the discretion of individual clinicians. Furthermore, an observational cohort design was adopted, which may not have controlled for unmeasured or unknown confounders that may have influenced the results. Second, epidural analgesia has not been evaluated for efficacy. Originally, cold tests or other methods should have been used to confirm the effectiveness of epidural analgesia, but this could not be done due to retrospective studies. However, the operating surgeon inserted the epidural catheter in this study while looking at the epidural space, and we estimate that the effect was uniform. Third, we were unable to establish NRS as a secondary outcome in this study. In our institution, the time point for assessing the analgesic effect of NRS was not fixed. It was not possible to determine whether the assessed NRS was a pre- or post-administration value of the analgesic. Therefore, we judged that we could not compare the NRS assessed at different time points. This point is an issue for the following prospective study. Forth, the duration of ICU stay may influence postoperative fentanyl consumption. Initially, a definite period should be set to define fentanyl consumption, but this study used the ICU electronic recording system. However, because the patients were of the same age and underwent the same surgery, there was no significant variation in the length of stay in the ICU (Table 1), and the use of electronic ICU records had the advantage of accurately recording opioid consumption. Fifth, the sample size is not adequately large to adjust for confounders. Our power analysis showed power = 0.34, which is insufficient power, and 462 cases were needed to achieve 80% power. However, the strengths of this study are that the sample size was more considerable than previous similar studies on AIS [10-12], and the fact that none of all studies could meet 80% power is a matter for future study. Sixth, because much of the information was collected manually, simple errors could have been introduced during data collection. Therefore, the work was divided among several individuals, with at least two individuals checking each piece of information. Finally, hyperalgesia and acute tolerance were converted to the outcome of opioid consumption in the ICU. Although this point has been discussed in previous studies [16], the evidence to date has replaced hyperalgesia and acute tolerance with the outcome of opioid consumption, which we believe is acceptable in this study.

## Conclusions

Intraoperative remifentanil dosage combined with propofol in surgery for AIS is not correlated with increased opioid consumption or other multimodal analgesic doses in the ICU, even in the setting of combined postoperative epidural analgesia. Owing to limitations in study design, these results require further investigation. Hence, well-designed prospective studies are warranted in the future.
